# Comparing the utility of mean versus intraindividual variability metrics in the relationship between mobility‐based digital phenotypes and cognition

**DOI:** 10.1002/alz.093386

**Published:** 2025-01-03

**Authors:** Katherine Hackett, Shiyun Xu, Moira McKniff, Ian Barnett, Tania Giovannetti

**Affiliations:** ^1^ Icahn School of Medicine at Mount Sinai, New York, NY USA; ^2^ University of Pennsylvania Perelman School of Medicine, Philadelphia, PA USA; ^3^ Temple University, Philadelphia, PA USA

## Abstract

**Background:**

Intraindividual variability (IIV) on neuropsychological task performance has demonstrated enhanced sensitivity to neuropathological decline compared to mean performance. It is currently unknown whether increased IIV in everyday behaviors may also lend added sensitivity to detect early subtle changes seen in pre‐clinical ADRD.

**Methods:**

In a pilot smartphone digital phenotyping study, 34 participants (M_age_ = 71.6±5.5; M_education_ = 16.4±2.7; 68% female 57% non‐Hispanic White) with healthy cognition or Mild Cognitive Impairment downloaded an open‐source smartphone application (mindLAMP) to passively capture GPS trajectories for 4 weeks. Monthly mean and day‐to‐day standard deviation (IIV) metrics for three GPS features were generated to test *a priori* hypotheses based on a conceptual framework. Correlations examined the relationship between mean versus IIV mobility metrics and 11 baseline neuropsychological T‐scores adjusted for age, sex, education, and estimated premorbid IQ. Hierarchical linear regressions examined whether mobility IIV was independently associated with cognition after adjusting for mean mobility, and vice versa, for relationships in which both mean and IIV were significantly correlated with the same cognitive outcome.

**Results:**

Greater mean GPS activity (radius of gyration), less mean GPS routine (physical circadian routine), and greater IIV in these two GPS features were both significantly associated with a neuropsychological measure of language (Boston Naming Test; .39≤|*r’s*|≤.55, *p*’s<.05). IIV in GPS routine did not explain a significant amount of additional variance in Boston Naming scores after controlling for mean GPS routine, whereas mean routine explained an additional 11% of variance after controlling for IIV (Overall model: R^2^ = .34, F(2,31) = 8.07, *p* = .002). Unique associations were identified between IIV in GPS routine and animal fluency scores (*r* = .44, *p*<.05) and IIV in GPS location diversity and Boston Naming (*r* = .35, *p*<.05), whereas mean metrics of these features were not associated with corresponding outcomes.

**Conclusions:**

Mobility‐based digital phenotyping features appear to be selectively associated with demographically‐corrected neuropsychological measures of language. Considering IIV in everyday mobility behaviors – in addition to overall mean mobility features ‐ may lend added value. Next steps will evaluate and compare a greater array of digital phenotyping features (mean and IIV) in predicting cognition and other relevant outcomes.

